# Robotic resection of a giant thymolipoma in a pediatric patient

**DOI:** 10.1093/jscr/rjae691

**Published:** 2024-11-07

**Authors:** Rae Hanke, Bryanna Emr, Matthew Taylor, Aodhnait S Fahy

**Affiliations:** Division of Pediatric Surgery, Penn State Health Children’s Hospital, 600 University Dr, Hershey, PA 17033, United States; Division of Pediatric Surgery, Penn State Health Children’s Hospital, 600 University Dr, Hershey, PA 17033, United States; Division of Thoracic Surgery, Penn State Milton S. Hershey Medical Center, 500 University Dr, Hershey, PA 17033, United States; Division of Pediatric Surgery, Penn State Health Children’s Hospital, 600 University Dr, Hershey, PA 17033, United States

**Keywords:** giant thymolipoma, robotic resection, pediatric, thymus

## Abstract

Thymolipomas are benign lesions in the anterior mediastinum that most commonly present in the first three decades of life. They often are asymptomatic and can be very large at time of diagnosis. After incidental detection of a thoracic mass on an abdominal ultrasound, an otherwise well 10 years old male was evaluated with further imaging, including a cardiac-gated magnetic resonance imaging (MRI) study. This demonstrated that the mass was intimate with but did not appear to invade the pericardium, likely originating from the thymus. Despite the large size, the patient underwent robotic resection of the mass and we include photographs illustrating the minimally invasive approach and highlighting critical structures. The patient tolerated the procedure well and recovered quickly. Final pathology was consistent with a giant thymolipoma. In summary, workup of giant thymolipomas is optimized with cardiac gated imaging. Despite their large size, these can be safely managed in a minimally invasive fashion in pediatric patients.

## Introduction

Thymolipomas are rare benign neoplasms with only around 200 cases reported in the literature. They account for 2%–9% of thymic neoplasms [1, 2]. They are usually asymptomatic and can be very large by the time of detection [3]. They can be confused on imaging studies with pulmonary sequestrations, cardiomegaly, or diaphragm eventrations or masses [4–7]. Surgical excision is curative and is the mainstay of care with no reported recurrences after resection. Secondary to their large size and location, most resections in pediatric patients in the literature have been accomplished through open thoracotomy or sternotomy [8–12]. There are two recent reports of thoracoscopic resection of a giant thymolipoma in pediatric patients [13, 14] and an adult report of successful robotic thymolipoma resection [15]. We report the detailed workup and operative management of a 10 year old patient with a giant thymolipoma who underwent a successful robotic resection. This case highlights key features of the workup as well as the potential for minimally invasive management of these masses which were traditionally resected in an open fashion.

## Case report

**Figure 1:**
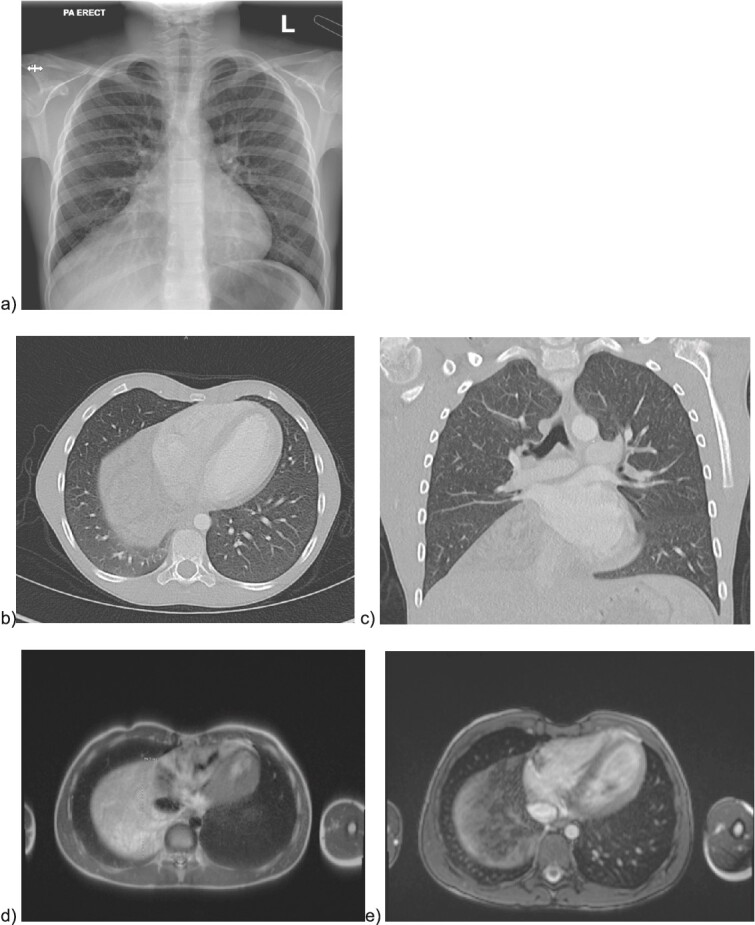
Preoperative imaging of the mediastinal mass. a) CXR illustrating the obscured right hemidiaphragm that was incidentally detected leading to further workup; b) axial and c) coronal CT images with intravenous contrast; e) MRI imaging with axial T2 HASTE sequence f) MRI axial VIBE sequence.

During a renal ultrasound to evaluate dysuria in a 10 year old male, an incidental mass was identified obscuring the right hemidiaphragm leading to further workup. He had no respiratory symptoms, though had reduced air entry on auscultation at the right base. Chest X ray (Fig. 1a) confirmed silhouetting of the right heart border. CT with intravenous contrast (Fig. 1b and c) showed a lower anterior mediastinal mass measuring 10 cm × 9 cm × 8 cm. It was heterogeneous and contained areas of fatty and soft tissue attenuation concerning for a teratoma or thymolipoma. Cardiac-gated chest MRI was then obtained which showed that the mass likely originated in the thymus and extended adjacent to the right atrial appendage and right atrium onto the diaphragm but did not invade pericardium or diaphragm (Fig. 1d and e). The signal was most consistent with a fatty tumor consistent with a giant thymolipoma.

**Figure 2:**
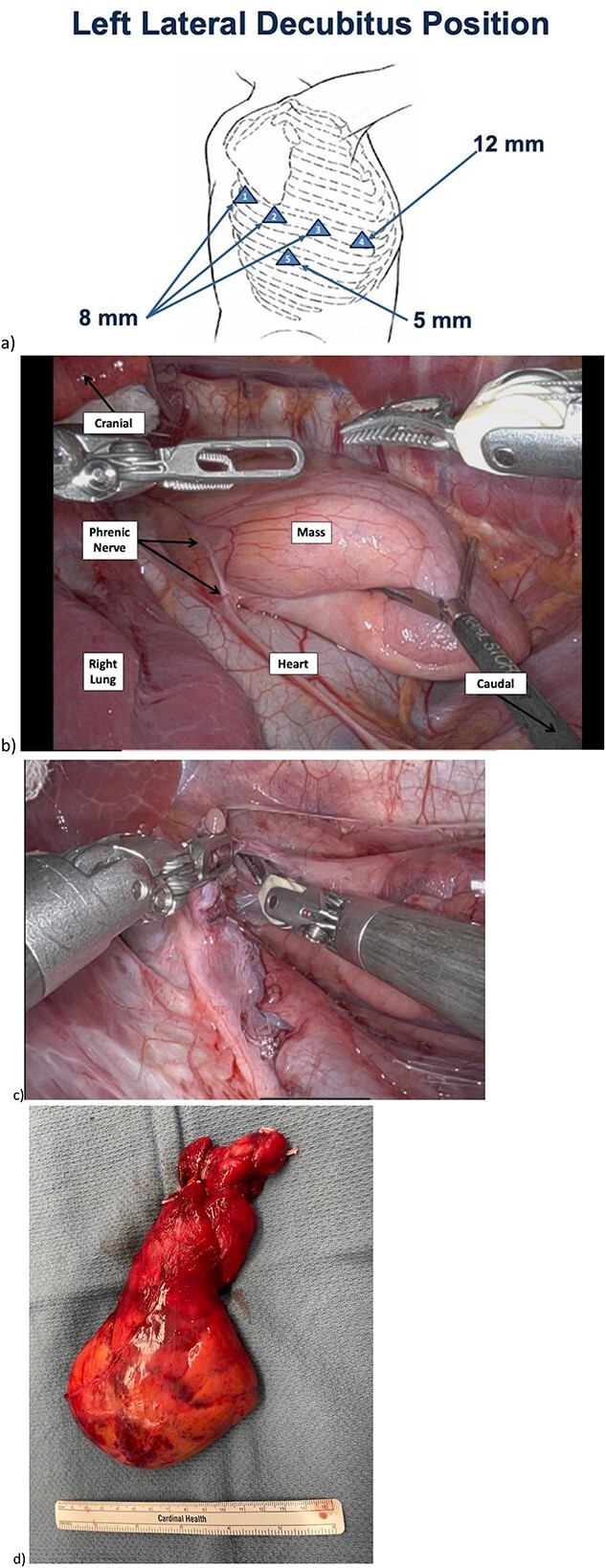
Intraoperative imaging of mediastinal mass dissection. a) Positioning and port placement. At completion the 12 mm port was upsized to 2.5 cm for specimen extraction. b) Prior to beginning resection, phrenic nerve easily visualized, mass overlying heart in usual thymic position. c) Cervical dissection with pedicles of two thymic vessels which were encircled and clipped. d) Resected mass.

Given concern for sampling error on tissue biopsy of such a large mass, the patient was offered upfront resection. The patient underwent double lumen endotracheal tube intubation to achieve single lung ventilation and was positioned in left lateral decubitus (port placement in Fig. 2a). The mass was draped over the pericardium, and we were able to clearly identify the phrenic nerve (Fig. 2b). We set up right lung retraction with a rolled gauze. We incised the pleura over the mass with bipolar cautery, taking care to avoid injuring the phrenic nerve. We dissected the pleura off the lesion and elevated it off the pericardium carefully, taking care to elevate it off the arch of the aorta and SVC superiorly. At the superior aspect of the mass, we identified distinct thymic veins and the thymic horns, each of which we dissected circumferentially and clipped with hemolock clips (Fig. 2c). We placed the mass in an endocatch bag and brought it out through the 12 mm port. We expanded this port by 1 cm to enable the mass to come out en bloc.

Recovery was uneventful and he discharged from the hospital on postoperative Day 3. Adequate pain control was achieved with tylenol, advil and lidocaine patches, with no need for narcotic medication. Pathology was consistent with a thymolipoma. He returned to competitive sports two weeks postoperatively. Followup MRI imaging at 1 and 2 years had no evidence of residual or recurrent disease.

## Discussion

We present the case of a robotic resection of an asymptomatic giant thymolipoma in a pediatric patient. We highlight features of the workup including the role for cardiac-gated MRI, and illustrate the potential benefits of minimally invasive resection of this large mass.

Thymolipomas are rare benign mediastinal tumors composed of thymic and mature adipose tissue which often present as diagnostic dilemmas and can be confused with pulmonary sequestrations, cardiomegaly, cardiac tumors, diaphragm eventration or diaphragm tumors on initial imaging. Diagnosis of thymolipomas is suggested by cross-sectional imaging illustrating a fatty appearing mass in the anterior mediastinum, contiguous with the thymus. We highlight that a cardiac-gated MRI can be helpful in determining if there’s a plane between the mass and the pericardium, offering reassurance that operative management will not require intracardiac dissection. Given the characteristic imaging, we elected to perform an upfront resection rather than obtain tissue for diagnosis. Given the large size of many giant thymolipomas, there are concerns about sampling error on percutaneous biopsies, and so in this case, we elected to forego preoperative cytological or histological evaluation.

Thymolipomas are classically well-circumscribed and encapsulated, with minimal adhesions or invasion into surrounding structures, and a pedicled vascular stalk. We highlight in the figures and video that robotic resection allows the operating surgeon to set up for superb exposure of the thymus and in this case, the thymolipoma. Identification of the phrenic nerve was easily achieved so that it can be protected throughout, and elevating the pleura off the mass then led to a smooth dissection. Exposure at the thoracic inlet can also be optimally achieved robotically, and this allowed for safe dissection of the thymic vessels and thymic horns. Most of the literature indicates that these lesions are managed with median sternotomies or thoracotomies as their size is often prohibitive to a more minimally invasive approach. Although open approaches via median sternotomy and lateral thoracotomies can also provide excellent exposure and control of vessels, minimally invasive resections can offer reduced recovery time, improved postoperative pain control, and fewer musculoskeletal concerns (for scoliosis or chest wall deformities) in pediatric patients. The fatty nature of the lesion means it was easily amenable to be manipulated out of the thoracic cavity through a small incision once it had been resected, allowing the patient to avoid a thoracotomy. In contrast to usual recovery after thoracotomy or sternotomy, the patient was discharged in a short time frame without need for narcotics and was able to return to competitive sports within two weeks of surgery.

## Conclusion

Workup for giant thymolipoma should include CT scan and cardiac-gated MRI to lead to the likely diagnosis. Surgical resection is the mainstay of therapy and can be achieved using minimally invasively even with a large tumor in a pediatric chest.
